# Varicella-Zoster Virus and the Eye: Clinical Spectrum, Management, and Vaccination

**DOI:** 10.3390/pathogens15020157

**Published:** 2026-02-02

**Authors:** Wendong Gu, Yaru Zou, Mingming Yang, Jing Zhang, Zizhen Ye, Jiaxin Deng, Yuan Zong, Kyoko Ohno-Matsui, Koju Kamoi

**Affiliations:** 1 Department of Ophthalmology & Visual Science, Graduate School of Medical and Dental Sciences, Institute of Science Tokyo, Tokyo 113-8510, Japan; gwd981107@gmail.com (W.G.); alicezouyaru519@gmail.com (Y.Z.); yangmm-12@outlook.com (M.Y.); zhangj.c@foxmail.com (J.Z.); yezizhen518@gmail.com (Z.Y.); dengjiaxin.med@gmail.com (J.D.); zongyuan666@gmail.com (Y.Z.); k.ohno.oph@tmd.ac.jp (K.O.-M.); 2 Department of Ophthalmology, Zhongshan Torch Development Zone People’s Hospital, Zhongshan 528436, China

**Keywords:** varicella-zoster virus, herpes zoster ophthalmicus, ocular inflammation, antiviral therapy, vaccination

## Abstract

Varicella-Zoster Virus (VZV) is one of the most important pathogens in ophthalmology. Reactivation may involve the adnexa (blepharoconjunctivitis, pseudomembranous conjunctivitis), cornea (dendritic keratitis, nummular and necrotizing stromal keratitis, disciform endotheliitis, neurotrophic ulcers, mucous-plaque keratitis) and sclera (episcleritis, anterior scleritis). Uveal inflammation ranges from anterior uveitis—with iris atrophy, trabeculitis-induced glaucoma and complicated cataract—to posterior necrotizing syndromes: acute retinal necrosis in immunocompetent hosts and progressive outer retinal necrosis in immunosuppressed patients, often complicated by occlusive vasculitis, macular edema, retinal detachment and phthisis. Optic nerve and cranial nerve involvement (optic neuritis, neuroretinitis, III/IV/VI palsies) and orbital inflammation may occur even without cutaneous signs (“zoster sine herpete”), making PCR-based intraocular diagnostics essential. Management relies on early, high-dose antivirals (acyclovir or valacyclovir), judicious corticosteroids and timely surgical intervention when required. Universal childhood varicella vaccination and recombinant zoster vaccination in adults ≥50 years have reduced VZV incidence and ocular complications in settings with high vaccine coverage, though rare post-vaccine keratitis or uveitis underscore the need for ongoing vigilance. In this review, we synthesize current knowledge on *varicella-zoster virus* ocular disease, with a focus on host–pathogen interactions that drive both injury and defense.

## 1. Introduction

Varicella-zoster virus (VZV, human herpesvirus 3) is the etiologic agent responsible for varicella and herpes zoster (HZ) [1]. VZV is one of the most ubiquitous human pathogens; as a neurotropic alpha herpesvirus, it infects most adults globally and persists in a latent state with lifelong risk of reactivation [2,3,4]. Primary infection usually occurs in childhood, presenting as varicella (chickenpox). After resolution of the acute illness, the virus builds lifelong latency in cranial nerve, dorsal root, and autonomic ganglia. With advancing age or immunosuppression, VZV can disrupt viral containment, resulting in reactivation and subsequent HZ [5,6].

Reactivation involving the ophthalmic division of the trigeminal nerve results in herpes zoster ophthalmicus (HZO), which accounts for approximately 10–20% of HZ cases [7,8]. HZO is clinically important because it carries the risk of a wide range of chronic complications, including persistent keratitis, recurrent anterior uveitis, scleritis, retinitis, and optic neuropathy, and leads to irreversible visual impairment [9,10,11]. In severe cases, these manifestations can result in permanent visual impairment, highlighting the significant disease burden of VZV on ocular health [12,13].

The pathogenesis of VZV-related ocular disease is complicated, involving direct viral cytopathic injury, dysregulated host immune responses, and VZV-induced vasculopathy [14]. These mechanisms give rise to a wide spectrum of ocular manifestations, affecting the anterior segment such as keratitis, uveitis, scleritis, and neurotrophic complications. Posterior segment involvement, including acute retinal necrosis (ARN) or progressive outer retinal necrosis (PORN), may also develop and represents the most vision-threatening end of the spectrum [9,15,16]. Despite advances in antiviral therapy and the availability of zoster vaccines, VZV-associated ocular disease remains a significant global concern. Recurrent inflammation, chronic pain, and structural complications contribute to long-term morbidity, highlighting the importance of early identification and timely intervention. Continued research on viral latency, host immune responses, and vaccine-induced immunity is expected to further improve our understanding and guide future therapeutic strategies [17,18,19].

This review aims to summarize current knowledge on VZV and its ocular manifestations, including its epidemiology, pathogenesis, clinical features, diagnostic approaches, and management. By integrating contemporary findings with recent advances, we highlight unresolved clinical and scientific issues and offer practical recommendations to support the diagnosis, management, and prevention of VZV-related ocular disease.

## 2. VZV Biology and Host–Pathogen Interactions

### 2.1. Virion Structure and Genome Organization

**Figure 1:**
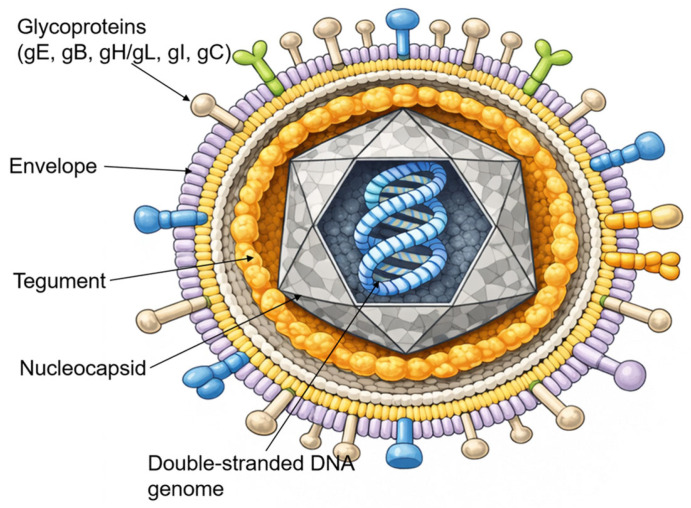
The VZV virion structure. Schematic representation of the VZV virion as an enveloped particle containing a linear double-stranded DNA genome enclosed within an icosahedral nucleocapsid, surrounded by a proteinaceous tegument and a host cell-derived lipid envelope bearing multiple viral glycoproteins (gE, gB, gH/gL, gI, and gC).

VZV is an enveloped, double-stranded DNA virus belonging to the *Alphaherpesvirinae* subfamily. It shares significant morphological and genomic homology with Herpes Simplex Virus (HSV) [1]. As illustrated in Figure 1, the mature virion is approximately 180–200 nm in diameter and consists of four distinct structural components: a central core containing the linear DNA genome, an icosahedral nucleocapsid, an amorphous proteinaceous tegument, and an outer lipid envelope derived from host cell membranes [20].

The viral envelope is studded with multiple glycoproteins, including glycoprotein E (gE), glycoprotein B (gB), glycoprotein H (gH), glycoprotein L (gL), glycoprotein I (gI), and glycoprotein C (gC), which mediate viral attachment, membrane fusion, and cell-to-cell spread. Among these, gE is the most abundant and immunogenic surface protein and plays a central role in viral replication and intercellular transmission [21].

The VZV genome is the smallest among the human herpesviruses, with a total length of approximately 125,000 base pairs (125 kbp). It is organized into two unique coding regions, the Unique Long (UL) and Unique Short (US) segments, each flanked by internal and terminal inverted repeats [3]. The genome encodes at least 71 open reading frames (ORFs), many of which are conserved orthologues of HSV genes. However, unlike HSV, VZV does not express a functional latency-associated transcript (LAT). Instead, viral latency and reactivation are regulated through distinct epigenetic mechanisms [22].

### 2.2. Viral Life Cycle and Latency in Sensory Ganglia

**Figure 2:**
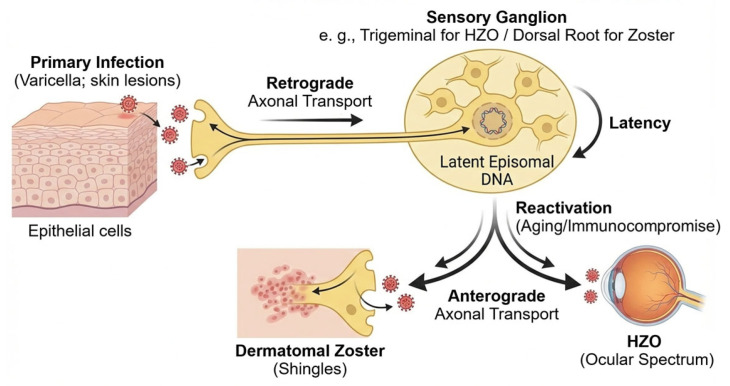
VZV life cycle and latency. After primary infection in epithelial cells (varicella), VZV enters sensory nerve terminals and is transported retrogradely to sensory ganglia (e.g., trigeminal or dorsal root ganglia). The virus then establishes lifelong latency in neuronal nuclei as circular episomal DNA with very limited transcription. Reactivation is favored by reduced cell-mediated immunity in older adults and in patients with chronic disease or immunocompromise. Reactivated virus returns anterogradely to peripheral tissues, causing dermatomal zoster and, when the ophthalmic division is involved, HZO with diverse ocular manifestations.

VZV is a neurotropic alpha herpesvirus that spreads from infected epithelial sites to the sensory neurons through retrograde axonal transport (Figure 2) [23]. After primary infection, the virus enters cranial and dorsal root ganglia and remains lifelong as circular episomes within neuronal nuclei [22]. In contrast to other alpha herpesviruses, VZV latency shows extremely limited transcription, with ORF63 as the major transcript, minor ORF62 activity, and the absence of infectious virions [3,22].

Neuronal defenses maintain latency through chromatin repression, autophagy, and baseline interferon-stimulated gene (ISG) activity. These neuronal mechanisms act together with viral proteins that reduce immune detection and support long-term persistence [23].

Reactivation occurs when cell-mediated immunity falls. This is most common in older adults, people with chronic disease, and immunocompromised hosts, where immunosenescence weakens VZV-specific T-cell memory [8]. Once active, the virus moves anterogradely to peripheral tissues and produces dermatomal zoster. Involvement of the ophthalmic division leads to a wide ocular spectrum, from epithelial keratitis to severe intraocular inflammation and retinal necrosis [24].

### 2.3. Mechanisms of Immune Evasion

**Figure 3:**
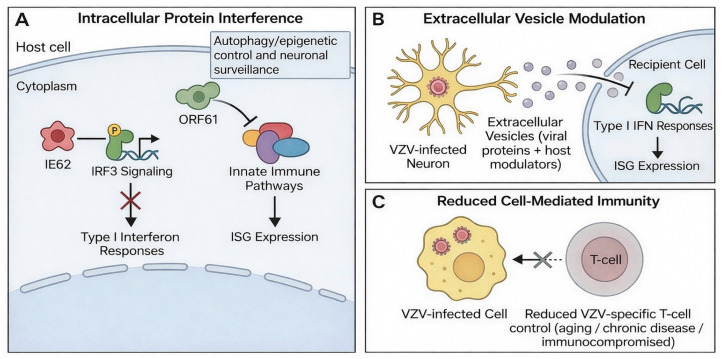
Mechanisms of VZV immune evasion. (**A**) Intracellular interference with innate immune signaling: IE62 inhibits IRF3 signaling to blunt type I interferon responses, while ORF61 disrupts innate immune pathways and reduces ISG expression. (**B**) Extracellular vesicle-mediated modulation: small extracellular vesicles released from VZV-infected neurons deliver viral and host immunomodulatory cargo to suppress type I interferon responses and alter ISG expression in recipient cells. (**C**) Reduced cell-mediated immunity (e.g., aging, chronic disease, or immunocompromise) decreases VZV-specific T-cell control, facilitating persistence and reactivation.

VZV uses multiple mechanisms to evade host antiviral defenses during primary infection, latency, and reactivation (Figure 3). IE62 interferes with IRF3 signaling and suppresses type I interferon responses, while ORF61 antagonizes innate immune signaling and reduces the expression of ISG [25,26] (Figure 3A). Other viral proteins alter autophagy, epigenetic control, and neuronal surveillance pathways, further weakening intrinsic antiviral barriers [23].

Recent studies describe an additional mechanism involving small extracellular vesicles released from VZV-infected neurons. These vesicles carry viral proteins and host immunomodulators that suppress interferon responses and modify antiviral gene expression in recipient cells [27] (Figure 3B). This long-range immunomodulation may increase susceptibility to secondary infections and contribute to neurologic and ocular complications [8].

These mechanisms allow VZV to maintain latency, enable reactivation when T-cell control decreases, and modulate inflammatory responses in neural and ocular tissues (Figure 3C).

### 2.4. Ocular Immune Responses and Tissue Injury

Reactivation of VZV in the trigeminal ganglion results in viral spread along the ophthalmic division to the cornea, uveal tract, and retina. Ocular injury arises from both direct viral cytopathy and secondary host-driven inflammation. Replication in corneal epithelial, stromal, or endothelial cells activates NF-κB and type I interferon responses, leading to epithelial keratitis, stromal keratitis, and anterior uveitis [28]. Inflammation in the trabecular meshwork may elevate intraocular pressure and lead to secondary glaucoma.

Electrophysiologic and imaging studies show bilateral changes in retinal ganglion cell function and corneal nerve morphology. Patients with herpes zoster ophthalmicus show reduced sub-basal nerve density in both eyes and abnormal pattern electroretinography, suggesting diffuse neuroimmune activation despite unilateral reactivation [29].

Stromal keratitis and recurrent anterior uveitis are associated with chronic inflammation and long-term visual loss. Their recurrence reflects the interaction of residual viral antigen, persistent immune activation, and dysregulated tissue repair pathways [24].

These findings indicate that VZV-related ocular diseases result from localized viral replication and immune dysregulation, with host inflammatory responses playing an important role in disease severity and chronicity.

## 3. Clinical Spectrum of VZV-Related Ocular Disease

VZV reactivation can involve almost every ocular structure, from the adnexa to the optic nerve. The main clinical manifestations by anatomical site are summarized in Table 1.

### 3.1. Adnexal and Conjunctival Manifestations

Adnexal involvement is common in HZO and may appear early in the course of reactivation [39]. Patients often show eyelid edema and vesicles along V1, and many develop secondary blepharitis from surface inflammation. Blepharoconjunctivitis is frequent in the acute phase and reflects direct viral involvement of the surface and secondary bacterial colonization. These adnexal manifestations seldom threaten vision directly but often precede or accompany deeper ocular disease [40,41].

**Figure 4:**
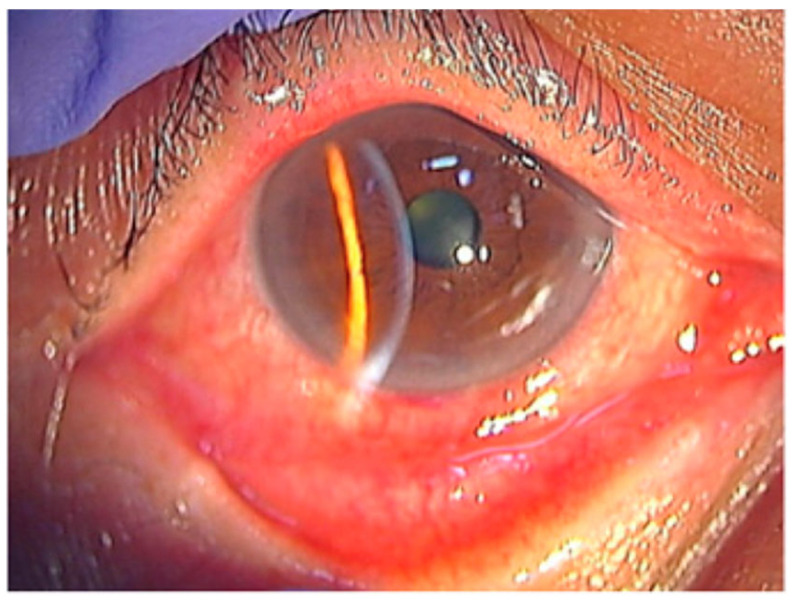
Conjunctival hyperemia and adnexal involvement in herpes zoster ophthalmicus (HZO). The picture shows marked conjunctival hyperemia with diffuse injection and mild chemosis, accompanied by eyelid edema, reflecting early surface involvement during VZV reactivation. Reproduced from Muto et al., Viruses, 2023, 15, 676, under the CC BY license [42].

Conjunctival inflammation is one of the most consistent early signs in HZO. Patients commonly have diffuse injection, mild chemosis, and a mixed follicular–papillary response (Figure 4) [42]. Studies show that conjunctival hyperemia is common, even in patients without overt skin lesions. This makes conjunctivitis helpful for identifying rash-negative HZO [31,43]. Pseudomembranes are less common. When present, they usually occur in severe epithelial inflammation or concurrent keratitis.

In early HZO, conjunctival and adnexal abnormalities often occur with early corneal surface changes, including coarse punctate epithelial lesions or small pseudodendrites [31,41]. These changes may appear before stromal or endothelial involvement and reflect viral cytopathy and early sensory nerve loss [39]. Recent reviews show that the adnexal and conjunctival findings are part of the disease spectrum and may help clinicians identify patients at risk of deeper ocular involvement [44].

In summary, adnexal and conjunctival findings play an important role in early HZO. These signs are usually mild but can mark the start of disease extension toward the cornea, uvea, or optic nerve. Careful examination and follow-up are therefore essential.

With progression of VZV-related surface inflammation, corneal involvement commonly develops and represents significant extension of adnexal and conjunctival disease.

### 3.2. Corneal Involvement

#### 3.2.1. Epithelial (Dendritic) Keratitis

**Figure 5:**
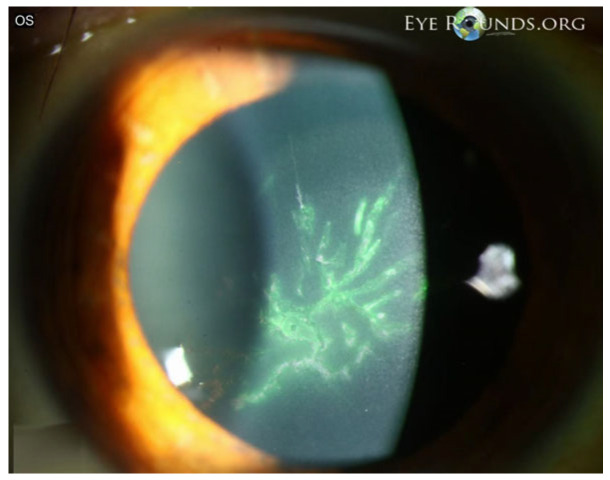
VZV epithelial keratitis presenting as pseudodendrites. The picture shows fluorescein-staining, elevated, irregular pseudodendritic epithelial lesions consistent with VZV epithelial keratitis. Courtesy of Welder J and Vislisel J, the University of Iowa, EyeRounds.org (Iowa City, IA, USA); used with the permission of the publisher [45].

Epithelial keratitis is often an early manifestation of VZV ocular involvement and reflects direct epithelial injury together with sensory dysfunction. In published clinical series and reviews, epithelial disease typically presents as coarse punctate lesions or pseudodendrites—elevated, irregular epithelial plaques that lack the terminal bulbs seen in herpes simplex and stain inconsistently (Figure 5) [45]. These early surface changes are frequently accompanied by eyelid or conjunctival inflammation and may precede stromal or endothelial involvement [28,46].

Several case series also describe epithelial irregularity and reduced corneal sensation during the acute phase of HZO, supporting the contribution of early neurotrophic changes to the presentation [47]. In the PCR-confirmed anterior segment series, epithelial abnormalities and conjunctival hyperemia were among the initial clinical signs, reinforcing the diagnostic value of early surface findings in VZV reactivation [31].

Most patients experience improvement with systemic antiviral therapy, and persistent epithelial instability is usually attributed to sensory nerve impairment rather than ongoing viral activity. Careful monitoring is recommended, as early epithelial disease may signal progression toward stromal keratitis, endotheliitis, or uveitis.

With ongoing immune activation or incomplete resolution, corneal involvement may extend beyond the epithelium into the stroma, resulting in immune-mediated stromal keratitis.

#### 3.2.2. Stromal Keratitis

Stromal keratitis is an immune-mediated form of corneal disease that often appears weeks to months after the acute phase of HZO. Patients with stromal infiltrates have a higher risk of recurrent inflammation, especially when corticosteroids are tapered. Although most cases occur in adults, pediatric stromal keratitis has also been described. A vaccinated child with discrete stromal opacities requiring prolonged topical steroids shows that immune-mediated stromal disease can occur in the pediatric population [48].

Most patients improve with combined antiviral and anti-inflammatory therapy, but the recurrent nature of the condition requires close monitoring and individualized medication adjustment.

#### 3.2.3. Endotheliitis and Disciform Keratitis

Endothelial involvement may cause sectoral or diffuse corneal edema, keratic precipitates, or short-term rises in intraocular pressure. These findings reflect viral activity along long ciliary nerves or local immune reactivation. Disciform edema has been reported in eyes with VZV-related anterior uveitis or posterior segment inflammation and shows the range of corneal manifestations linked to VZV [49]. Aqueous PCR testing is recommended in persistent or atypical cases to confirm viral involvement and guide treatment.

#### 3.2.4. Neurotrophic and Mucous-Plaque Keratitis

Neurotrophic keratitis typically emerges as a late sequela of VZV reactivation, when trigeminal nerve function is reduced to low corneal sensation and slow epithelial healing. Other studies show that unilateral HZO can lead to bilateral loss of subbasal nerves, leading to persistent epithelial instability [50]. Confocal microscopy findings also show nerve fiber reduction in both affected and fellow eyes, confirming that corneal denervation is common after HZO [51].

Mucous-plaque keratitis represents a chronic surface disturbance characterized by adherent mucus over irregular epithelium and is often associated with longstanding neurotrophic changes rather than active viral replication [52,53].

These conditions result from VZV-related nerve injury and usually require long-term lubrication, surface protection, and careful use of anti-inflammatory therapy.

### 3.3. Scleral Disease (Episcleritis, Scleritis)

Scleritis is an uncommon but clinically significant manifestation of VZV reactivation. It typically develops weeks to months after the acute episode and may occur in association with keratitis or anterior uveitis. Polymerase chain reaction (PCR) testing has demonstrated VZV DNA in aqueous humor samples from affected eyes, confirming that viral replication can directly involve the sclera. A representative case of PCR-positive nodular scleritis showed partial improvement with corticosteroids but required adjunctive antiviral therapy for complete resolution, supporting a mixed viral and immune-mediated pathophysiology [32,33].

These findings highlight the importance of considering antiviral coverage alongside anti-inflammatory treatment in selected patients and underscore the need for long-term follow-up in individuals with VZV-related ocular inflammation.

As inflammation extends intraocularly, uveal involvement becomes a prominent manifestation of VZV reactivation.

### 3.4. Uveal Inflammation

#### 3.4.1. Anterior Uveitis and Secondary Glaucoma

Anterior uveitis is a common intraocular sign of VZV reactivation. Patients may show anterior chamber inflammation, keratic precipitates, sectoral iris atrophy, and variable elevation in intraocular pressure [28,31]. In PCR-confirmed cases, uveitis often occurs in combination with endotheliitis and trabeculitis, indicating viral involvement of the corneal endothelium and aqueous outflow pathways [31].

Secondary glaucoma develops when inflammation affects the trabecular meshwork. Several clinical reports describe acute rises in intraocular pressure (IOP). One showed trabeculitis with IOP elevation to 50 mmHg, accompanied by corneal edema, diffuse keratic precipitates, and nasal iris atrophy five days after YAG iridotomy [54]. IOP improved with systemic antiviral therapy, corticosteroids, cycloplegia, and pressure-lowering treatment, demonstrating the need for simultaneous control of inflammation and IOP.

Sectoral iris atrophy reflects focal ischemic injury to the iris stroma and pigment epithelium [55]. Recurrent inflammation is more common in older or immunosuppressed individuals and often requires a slow corticosteroid taper [28]. Laser procedures may trigger reactivation in predisposed individuals, supporting prophylaxis in selected patients [54].

While anterior uveitis represents the most common intraocular presentation, VZV can also involve the posterior segment, leading to severe necrotizing syndromes.

#### 3.4.2. Posterior Necrotizing Syndromes

**Figure 6:**
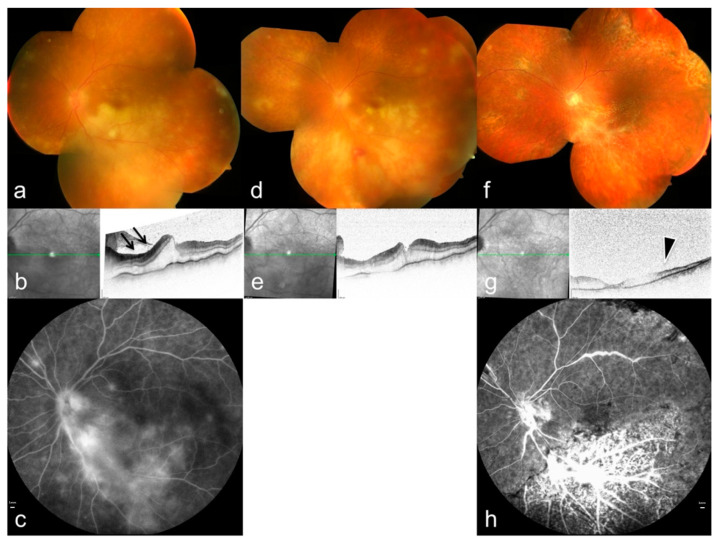
Multimodal imaging features of ARN caused by *varicella-zoster virus*. (**a**,**d**,**f**) Color fundus photographs (OCT) demonstrate multifocal peripheral retinal infiltrations and progressive circumferential involvement. (**b**,**e**,**g**) OCT shows ischemic changes in the inner retinal layers (arrows) with associated retinal edema; green lines indicate the OCT B-scan position. (**c**,**h**) Fluorescein angiography reveals occlusive vasculitis and extensive vascular leakage (arrowheads), reflecting severe inflammatory and ischemic damage. Reproduced from Mayer et al., Diagnostics, 2022, 12, 386, under the CC BY license [34].

ARN is the most severe posterior manifestation of VZV infection. It presents with multifocal peripheral retinitis, occlusive vasculitis, dense vitritis, and rapid circumferential necrosis (Figure 6) [34,56]. VZV is the main cause of ARN in immunocompetent patients. Several studies demonstrate increased MMP-3 and decreased TIMP-1 levels in VZV-associated ARN, suggesting that matrix imbalance may contribute to necrotic expansion [57].

Case reports illustrate variations in disease severity. ARN has occurred during PD-1 inhibitor therapy, with unusually rapid progression and extensive vascular involvement [58]. Additional cases emphasize that systemic immune alterations may influence the clinical course [59]. These findings highlight the importance of host immune status as a modifier of VZV retinitis severity.

Posterior uveitis with vitritis or subtle choroidal lesions may precede overt necrosis, emphasizing the importance of early recognition. PORN is a rare but aggressive variant seen mainly in severely immunocompromised patients. It features minimal vitritis, early macular involvement, and extremely rapid tissue destruction [46]. Both ARN and PORN carry a high risk of retinal detachment and severe visual loss, making immediate systemic antiviral therapy, with intravitreal treatment when needed [56].

In addition to retinitis, VZV is increasingly recognized as a cause of retinal and choroidal vasculopathy, which may occur independently or in association with necrotizing disease.

### 3.5. Retinal and Choroidal Vasculopathy

#### 3.5.1. Occlusive Retinal Vasculitis

Occlusive retinal vasculitis associated with VZV shows segmental arterial or venous narrowing, vascular sheathing, intraretinal hemorrhage, and areas of peripheral nonperfusion. Fluorescein angiography often reveals delayed arterial filling, capillary dropout, and vessel-wall leakage, consistent with inflammatory endothelial injury [36,60].

Some patients develop vascular occlusion before clear signs of retinitis, indicating that primary vascular involvement can start earlier than the necrotic phase [61]. Mechanistic analyses support a combination of direct viral invasion of vascular endothelium and secondary immune-mediated thrombogenic responses in the development of occlusion [60].

#### 3.5.2. Multifocal Chorioretinitis

Multifocal chorioretinitis occurs more often in pediatric or younger patients. It presents with discrete choroidal or outer retinal lesions, sometimes with mild vitritis. These lesions may appear before, or independently of, overt retinitis and represent localized choroidal inflammation [62,63]. Studies show focal hyperreflectivity of the outer retina and variable RPE involvement, supporting a primary choroidal process. Early antiviral therapy is recommended to prevent posterior extension or secondary complications.

#### 3.5.3. Choroidal Depigmentation and Structural Alterations

Sectoral or diffuse choroidal depigmentation may follow VZV reactivation. Reports describe hypopigmented choroidal patches corresponding to areas of thinning or stromal irregularity on OCT [64,65]. Enhanced-depth imaging further shows choroidal vascular attenuation or stromal remodeling, which suggests a chronic post-inflammatory process rather than active viral replication [66]. These structural alterations can persist even after the acute episode resolves.

#### 3.5.4. Immune-Mediated Choroidopathy

Immune-mediated choroidopathy after VZV infection may resemble birdshot-like or non-necrotizing multifocal choroidopathies. These studies demonstrate multiple hypopigmented choroidal lesions, mild vitritis and preserved retinal layers, without the rapid retinal destruction seen in necrotizing retinitis [67]. Studies further show that VZV can trigger diffuse vasculopathic and immune-mediated responses in both cerebral and ocular vessels, supporting the concept of a broader post-infectious inflammatory phenotype [68].

Beyond retinal and choroidal pathology, VZV can affect the optic nerve, cranial nerves, and orbital tissues, giving rise to a diverse spectrum of neuro-ophthalmic and orbital complications.

### 3.6. Neuro-Ophthalmic and Orbital Complications

#### 3.6.1. Optic Neuritis and Neuroretinitis

Optic nerve diseases in VZV infection may present as optic neuritis, neuroretinitis, or ischemic optic neuropathy. Patients often present with sudden visual loss, relative afferent pupillary defect, and optic disc edema. These signs may develop independently of cutaneous rash. Several reports document optic neuritis in “zoster sine herpete” should remain a differential consideration in acute unilateral optic neuropathy [69]. Studies also show that VZV can reach the optic nerve by spreading along trigeminal axons or by entering the bloodstream and causing secondary vasculopathy that affects the posterior ciliary circulation [32].

Neuroretinitis is reported with optic disc edema and macular star formation. Some patients develop partial cranial nerve palsies or early orbital inflammation at the same time, showing that VZV can involve several neurovascular structures at once [70]. Imaging shows optic nerve enhancement or perineural inflammation. Visual outcomes vary and depend on early treatment. Systemic antiviral therapy remains the mainstay, with corticosteroids used selectively to reduce inflammatory edema after viral replication is controlled.

#### 3.6.2. Cranial Nerve Palsies and Apex Syndromes

Cranial nerve involvement is a well-recognized complication of VZV, most affecting cranial nerves III, IV, and VI. Third-nerve palsy causes ptosis, diplopia, and loss of adduction, sometimes accompanied by optic neuritis or uveitis. Studies show that such combined neuropathies after HZO often occur in cases with delayed or inadequate early treatment [71,72]. Current evidence suggests that these neuropathies arise from inflammatory neuritis or ischemia caused by VZV-related vasculopathy [32].

Orbital apex syndrome represents a more severe pattern. It causes visual loss, ophthalmoplegia, reduced corneal sensation, and imaging signs of posterior orbital inflammation. One detailed case showed orbital apex involvement with superior ophthalmic vein thrombosis, illustrating that VZV can produce both inflammation and thrombosis in the confined apex region. Other reports show apex inflammation emerging from HZO-associated uveitis or from presentations that resemble bacterial orbital cellulitis [73]. Early systemic antivirals and corticosteroids, with exclusion of bacterial infection, are essential to minimize irreversible visual loss.

#### 3.6.3. Orbital Myositis and Cellulitis-like Presentations

Orbital myositis may occur early in VZV reactivation. It can appear before skin eruption. Studies show painful ophthalmoplegia with imaging evidence of selective enlargement and increased signal of affected extraocular muscles, consistent with focal inflammatory myositis. This presentation is reported in both adults and adolescents and may be mistaken for idiopathic orbital inflammatory syndrome or cellulitis-like presentations [38,74]. Muscle involvement likely reflects localized viral-induced inflammation or secondary immune injury.

More severe patterns resemble orbital cellulitis or phlegmon. One case showed progressive orbital swelling and major visual decline, which required urgent systemic therapy [75]. Another series described diffuse preseptal and postseptal inflammation mimicking bacterial cellulitis despite negative cultures [74]. These reports indicate that VZV may involve deeper orbital structures, particularly in older or immunocompromised patients. Recognition of VZV as a potential etiology is critical, as antiviral therapy is required to prevent irreversible structural and functional damage.

## 4. Diagnostic Strategies

### 4.1. Clinical Examination and Imaging

Diagnosis of VZV-related ocular disease requires repeated assessment because findings change over time. Early signs often start at the eyelids, conjunctiva, and corneal surface. Studies report that early VZV ocular involvement includes pseudodendrites, coarse punctate epithelial erosions, and a follicular conjunctival reaction [40]. Sectoral iris atrophy is an important diagnostic marker, reflecting localized ischemia from vasculopathy and direct neural involvement, a pattern repeatedly described in modern anterior uveitis literature [11].

Posterior involvement needs the same attention. Widefield imaging and OCT have improved early detection of necrotizing retinitis. Imaging-based ARN cohorts show sharply outlined areas of necrosis, arterial inflammation with capillary dropout, loss of the ellipsoid zone, and fast centrifugal extension of lesions. These patterns match the known mechanisms of VZV-mediated vasculitis [34,76]. Autofluorescence can reveal subclinical extension, while OCT allows measurement of photoreceptor damage and optic nerve swelling that may guide prognosis [77,78]. Anterior segment imaging, including in vivo confocal microscopy, may provide additional information on corneal nerve loss and inflammatory changes but is generally used as an adjunctive tool in selected cases rather than as a routine diagnostic modality [51].

**Figure 7:**
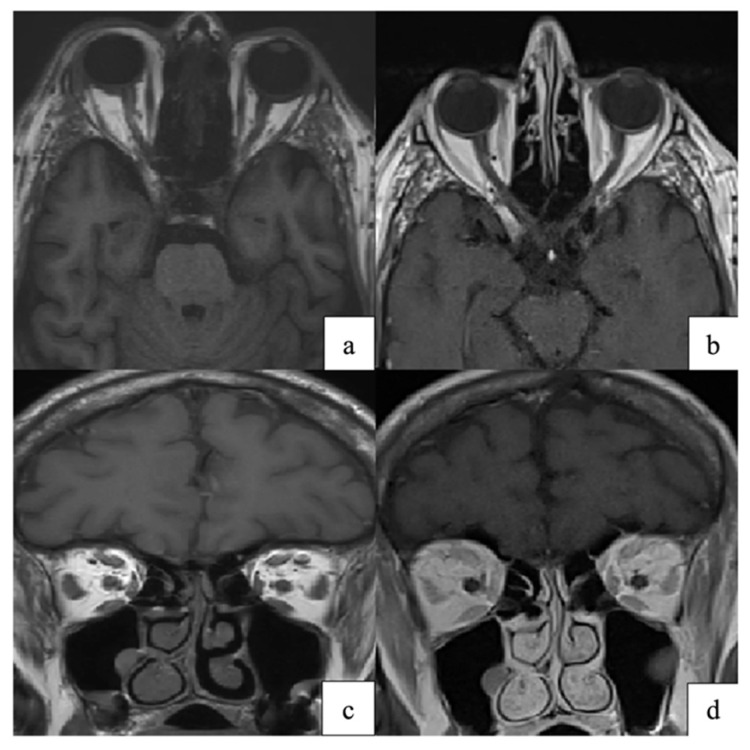
(**a**,**c**) Axial and coronal T1-weighted orbital MRI showing altered signal intensity and enlargement of the right superior rectus, medial rectus, lateral rectus, and superior oblique muscles, consistent with orbital myositis following herpes zoster ophthalmicus. (**b**,**d**) At one-month follow-up, axial and coronal T1-weighted images demonstrate marked improvement of extraocular muscle myositis. Reproduced from Pace et al., Pathogens 2024, 13, 832, under the CC BY license [79].

Neuro-ophthalmic disease adds diagnostic complexity. MRI may show optic nerve enhancement, intraconal fat infiltration, or extraocular muscle enlargement, reflecting VZV-related neuritis or orbital myositis (Figure 7) [79]. These imaging patterns are consistent with proposed mechanisms of VZV spread and injury, including transaxonal spread, vasculopathy, and immune-mediated injury [8,80].

Overall, multimodal imaging provides structural, functional, and vascular information that complements clinical examination and improves early detection of sight-threatening VZV disease. The principal diagnostic tools and their roles in VZV-related ocular disease are summarized in Table 2.

### 4.2. Laboratory Confirmation (PCR, Intraocular Antibodies)

Molecular diagnostics is essential for confirming VZV infection, particularly in atypical or posterior segment presentations. PCR of aqueous or vitreous samples offers high sensitivity and specificity. Multiplex PCR panels help distinguish VZV from HSV-1, HSV-2, or CMV, which is important when evaluating necrotizing retinitis with overlapping features [81,82,83,84,85,86,87,88,89]. Experimental work also shows that VZV can suppress innate immune signaling through extracellular vesicle-associated viral components, which may affect early diagnostic timing [8,23].

In later or partially treated disease, intraocular antibody detection becomes useful. VZV-specific IgG measured by the Goldmann–Witmer coefficient helps diagnose chronic anterior uveitis, late ARN, and PCR-negative cases. Immune studies show persistent intraocular antibody production after VZV reactivation, a pattern also seen in VZV encephalitis and vasculitis [8,27].

Neurological involvement requires broader evaluation. CSF PCR and CSF anti-VZV IgG support diagnosis of optic neuritis, cranial neuropathies, or meningitis, particularly in zoster sine herpete. These assays align with known pathways of VZV spread, including transaxonal and hematogenous dissemination [2,80].

Central nervous system evaluation is indicated in patients with neuro-ophthalmic or neurological manifestations. By combining imaging, molecular testing, and immunological assays, clinicians can diagnose VZV across its wide clinical spectrum—from isolated epithelial disease to necrotizing retinitis and neuro-ophthalmic involvement.

## 5. Therapeutic Management

### 5.1. Antiviral Regimens

Systemic antiviral therapy remains the core treatment for acute HZO. Clinical studies and guideline-based recommendations show that early treatment—ideally within the first 72 h—reduces viral replication and speeds skin and ocular recovery [90,91]. Early use also lowers the risk of complications such as epithelial keratitis, stromal keratitis, and hypertensive anterior uveitis. Acyclovir (800 mg five times daily), valacyclovir (1000 mg three times daily), and famciclovir are considered equivalent first-line choices for immunocompetent patients, with valacyclovir offering better bioavailability and simpler dosing.

Short courses remain the standard for acute disease. Valacyclovir, acyclovir, and famciclovir are effective when started early and reduce the risk of epithelial keratitis, stromal disease, and uveitis. Long-term suppression remains an area of active study [92]. A recent randomized clinical trial from the Zoster Eye Disease Study (ZEDS) evaluated low-dose valacyclovir for one year in patients with HZO-related postherpetic neuralgia. The primary endpoint was negative, but secondary analyses showed less pain, shorter pain duration, and lower analgesic medication use in selected patients, particularly those younger than 60 years with chronic symptoms [93].

Intravenous acyclovir is preferred in immunocompromised patients and in severe posterior involvement such as ARN. Modern ARN series emphasize the need for rapid systemic therapy, with intravitreal antiviral injections used as an adjunct to achieve prompt intraocular viral suppression rather than as a substitute for systemic treatment [81].

### 5.2. Role of Corticosteroids

Systemic corticosteroids may be considered in severe scleritis, optic neuritis, or orbital inflammation but should only be introduced after antiviral therapy has been initiated. For epithelial keratitis, clinicians usually avoid steroids until the surface heals. In contrast, stromal keratitis and disciform endotheliitis often require topical corticosteroids to reduce inflammation and prevent scarring. Clinical cohorts show that steroids work best when patients receive antiviral coverage at the same time to avoid prolonged viral activity [94,95].

In HZO-associated anterior uveitis, topical steroids lower inflammation and reduce the risk of posterior synechiae and pressure elevation from trabeculitis. Older adults often show prolonged or recurrent inflammation and may need a slow steroid taper to avoid relapse [55]. Pressure-lowering drops are usually effective, but a short systemic agent may be required for severe spikes.

Systemic corticosteroids may help in severe scleritis, optic neuritis, cranial neuropathies, or orbital inflammation. Neuro-ophthalmic cases show good responses when systemic steroids accompany antiviral therapy, supporting a combined inflammatory and vascular mechanism [8,80].

### 5.3. Surgical Interventions and Complication Management

Surgical interventions are reserved for complications that threaten vision or result from structural damage. Neurotrophic keratopathy may require tarsorrhaphy or amniotic membrane transplantation, with cenegermin considered in refractory cases [94,96]. Persistent epithelial defects are common in patients with reduced corneal sensation and require aggressive ocular surface rehabilitation [80,97].

Patients with elevated intraocular pressure improve with medical therapy. A smaller group may progress to secondary glaucoma and require laser or surgery [55]. Reports stress early pressure control to protect the optic nerve, particularly in older adults with repeated episodes [80,96,97].

ARN carries a high risk of retinal detachment. Several reports support early pars plana vitrectomy in selected patients, especially those with large areas of necrosis or early traction. This approach may lower the risk of retinal detachment and help preserve vision [81,98]. Barrier laser can be used around active necrotic borders, though evidence on its benefit remains mixed.

Orbital complications such as myositis or apex involvement may require decompression or biopsy when vision is threatened or diagnosis is unclear. MRI usually guides these decisions, and management often requires input from multiple specialties [29,99].

## 6. Vaccination: Prevention and Ocular Safety

### 6.1. Varicella Vaccination in Childhood

Childhood varicella vaccination has greatly reduced primary VZV infection, hospitalization rates, and severe complications. Large population studies show significant declines in varicella incidence after the introduction of two-dose programs, along with reduced household transmission and strengthened herd immunity [100,101]. Immunologic studies indicate that childhood vaccination induces durable antibody and T-cell responses, which suppress viral replication and contribute to long-term protection [102,103].

Early-life vaccination also decreases community circulation of VZV. This offers indirect protection to adults and immunocompromised individuals [104]. Public health and modeling analyses show that vaccination lowers the lifelong risk of herpes zoster and related ocular disease by reducing opportunities for primary exposure and modifying the epidemiologic patterns of viral latency and reactivation (breaking the cycle, life-course vaccination strategy). Childhood vaccination remains a key measure for preventing primary infection and reducing later ophthalmic morbidity [90,100].

### 6.2. Zoster Vaccines in Adults

Adult vaccination focuses on preventing VZV reactivation and its complications, including HZO. The live-attenuated zoster vaccine (ZVL) provides moderate protection that diminishes with age because of a decline in T-cell-mediated immunity. The recombinant subunit vaccine (RZV) induces strong and sustained CD4^+^ T-cell responses through its AS01B adjuvant system and maintains high efficacy across age groups and in individuals with chronic conditions [21,105].

Long-term studies show that RZV significantly reduces the incidence of herpes zoster, post-herpetic neuralgia, and ophthalmic involvement more effectively than ZVL or no vaccination [106,107]. Immunologic data confirm that RZV enhances antigen presentation and supports central memory T-cell persistence, helping counteract age-associated declines in cellular immunity [14,21]. Health-economic evaluations identify RZV as the most cost-effective strategy because it provides durable protection and reduces visual morbidity from HZO. Current guidelines therefore recommend RZV as the preferred vaccine for adults. However, vaccination strategies require additional consideration in immunocompromised individuals, who are at particularly high risk for severe VZV-related ocular disease.

Immunocompromised patients are at increased risk for severe VZV-related ocular disease, including rapidly progressive conditions such as ARN and PORN [41,76]. Given these considerations, zoster vaccination requires special attention. ZVL is contraindicated in most immunosuppressive settings, whereas the RZV is preferred because it does not contain live virus [14,108]. However, immune responses to vaccination may be reduced in immunocompromised individuals, and current evidence remains limited regarding the effectiveness of vaccination in preventing severe ocular disease [14]. This remains an important area for future research.

### 6.3. Rare Post-Vaccine Ocular Events

Although vaccines are generally safe and well-tolerated, ocular adverse events can occasionally occur following immunization [109,110,111,112,113]. VZV vaccines have favorable safety profiles, although rare ocular inflammatory events have been reported following either varicella or zoster vaccination [114]. Case reports describe occasional instances of anterior uveitis, stromal keratitis, and optic neuritis emerging shortly after vaccination, with most cases responding well to antiviral therapy and corticosteroids [92,115]. Large surveillance studies show that these events are extremely uncommon, and no causal link has been confirmed [116].

By contrast, natural VZV reactivation carries a much higher risk of ocular complications, including keratitis, uveitis, and acute retinal necrosis. These complications occur far more frequently than the isolated events reported after vaccination [1,117]. Immunologic studies further show that vaccination improves viral control and therefore reduces reactivation-related ophthalmic disease [18,105].

Overall, current evidence supports the safety and effectiveness of both childhood varicella vaccination and adult zoster vaccination. RZV provides the most durable immune protection and the most favorable risk–benefit profile for preventing ocular disease. An overview of the main therapeutic approaches for VZV-related ocular disease is provided in Table 3.

## 7. Future Directions and Research Priorities

Major progress has improved current understanding of VZV biology, but several gaps remain. Basic science work still needs a clear map of latent viral transcription in human sensory ganglia, especially the role of low-level gene expression and the neuronal pathways that keep latency stable or allow it to fail [1,3,22,23]. Studies of viral immune evasion, such as extracellular vesicles released from infected neurons, cytokine modulation, and neuron–immune signaling, may help identify new therapeutic targets [25,26,27].

Clinical research priorities include defining long-term outcomes and identifying predictors of chronic inflammation, recurrent keratitis, and secondary glaucoma [96]. Large natural-history studies and biomarker research may allow earlier recognition of high-risk disease and guide individualized antiviral or anti-inflammatory therapy [123]. Advances in imaging, including high-resolution OCT and widefield angiography, should be evaluated for detecting subclinical vasculopathy, early retinitis, or early neurotrophic changes. These tools may improve prognostic assessment and allow earlier intervention [77,78].

Therapeutic trials remain a major unmet need. Recent evidence from the Zoster Eye Disease Study (ZEDS) did not show reduced postherpetic neuralgia with one year of valacyclovir, but secondary outcomes suggested meaningful benefit in selected patients, including reduced pain burden and lower medication needs [93]. These findings highlight the importance of defining which clinical subgroups respond to long-term antiviral suppression and the need for controlled trials focusing on recurrent keratitis, uveitis, and neurotrophic disease [27,31,93].

Finally, vaccine-related investigations should extend beyond epidemiologic monitoring to assess vaccine performance across different age groups, immunocompromised populations, and individuals with prior ocular involvement. Understanding the long-term effect of childhood varicella vaccination on lifetime risk of HZO and ocular complications remains important as vaccinated cohorts age.

## 8. Conclusions

VZV remains a major cause of ocular morbidity worldwide, with disease manifestations spanning the adnexa, cornea, uveal tract, retina, and optic nerve. Recent work has clarified key aspects of viral latency, immune evasion, and host responses, while improved diagnostic tools and therapeutic approaches have strengthened clinical management. However, recurrent inflammation, chronic keratitis, and secondary glaucoma remain common, particularly in older adults and immunocompromised individuals.

Early recognition and prompt antiviral and anti-inflammatory treatment are essential to prevent irreversible visual loss. Emerging evidence suggests a role for prolonged antiviral suppression in selected patients with recurrent keratitis or uveitis, although further trials are required to refine who benefits and how long treatment should continue. Prevention remains central to reducing disease burden. Universal childhood varicella vaccination and widespread use of recombinant zoster vaccine in adults have reduced both primary infection and reactivation-related eye disease.

Future progress will depend on the continued integration of basic, translational, and clinical research. Improved imaging, better molecular diagnostics, and more targeted therapies may allow earlier detection and more personalized treatment. As patterns of VZV infection change with broader vaccine coverage, continued monitoring and ongoing research will be necessary to improve outcomes for patients with VZV-related ocular disease.

## Figures and Tables

**Table 1:** Clinical spectrum of varicella-zoster virus-related ocular disease by anatomical site.

Anatomical Site	Typical Manifestations	Key Clinical Features
Adnexa and conjunctiva	Eyelid vesicles, eyelid edema, blepharitis, follicular or mixed conjunctivitis	Common in early HZO and may precede intraocular disease; careful examination of the V1 dermatome and conjunctiva is important [10].
Corneal epithelium	Pseudodendritic epithelial keratitis, punctate epithelial erosions	Often an early sign of ocular VZV reactivation; pseudodendrites are elevated, irregular, and lack terminal bulbs, and may appear before stromal or endothelial involvement [30].
Corneal stroma and endothelium	Stromal keratitis, nummular infiltrates, endotheliitis, disciform keratitis	Predominantly immune-mediated; frequently associated with anterior uveitis and raised intraocular pressure, and can be recurrent, steroid-responsive, and steroid-dependent [31].
Neurotrophic cornea and late surface disease	Neurotrophic keratopathy, persistent epithelial defects, mucous-plaque keratitis	Reflects trigeminal denervation and reduced corneal sensitivity; often occurs as a late sequela and requires intensive ocular surface support and, in some cases, surgical procedures [15].
Sclera	Episcleritis, anterior scleritis (diffuse or nodular)	Uncommon but potentially sight-threatening; may develop weeks to months after HZO, often with concomitant keratitis or uveitis, and usually requires systemic anti-inflammatory therapy in addition to antivirals [32,33].
Anterior uvea	Anterior uveitis with keratic precipitates, sectoral iris atrophy, secondary glaucoma	One of the most frequent intraocular manifestations; characterized by recurrent inflammation and episodes of markedly elevated intraocular pressure, typically attributed to trabeculitis [10,15].
Posterior uvea and retina	Acute retinal necrosis, progressive outer retinal necrosis, multifocal chorioretinitis	Necrotizing retinitis with occlusive vasculitis and vitritis in immunocompetent hosts (ARN) and rapidly PORN with minimal inflammation in severely immunosuppressed patients [34,35].
Retinal vasculature	Occlusive retinal vasculitis, peripheral non-perfusion, ischemic complications	Arteriolar and venous narrowing, vascular sheathing, hemorrhages, and capillary drop-out; fluorescein angiography is essential to detect non-perfusion and to guide management [36].
Optic nerve and cranial nerves	Optic neuritis, neuroretinitis, third, fourth, or sixth nerve palsy	May occur with or without a skin rash, including in zoster sine herpete; neuro-ophthalmic involvement often warrants neuroimaging and central nervous system evaluation [37].
Orbit	Orbital myositis, orbital inflammatory syndrome, cellulitis-like presentations	Can mimic idiopathic orbital inflammation or bacterial cellulitis; recognition of VZV as the cause is important to ensure timely antiviral therapy [38].

Abbreviations: VZV, varicella-zoster virus; HZO, herpes zoster ophthalmicus; V1, ophthalmic division of the trigeminal nerve; ARN, acute retinal necrosis; PORN, progressive outer retinal necrosis.

**Table 2:** Diagnostic tools for varicella-zoster virus-related ocular disease.

Diagnostic Modality	Primary Role	Key Points
Clinical examination	Initial recognition of HZO and ocular involvement	Includes inspection of the V1 dermatome, slit-lamp examination of the ocular surface and anterior segment, and dilated fundus examination to detect early intraocular disease [40].
Multimodal retinal imaging (fundus photography, OCT, fluorescein angiography)	Structural and vascular assessment of posterior segment disease	Widefield or conventional fundus imaging documents necrotizing retinitis and peripheral non-perfusion; OCT detects retinal thinning, macular edema, and optic disc swelling; fluorescein angiography reveals occlusive vasculitis, leakage, and capillary drop-out in vasculopathy and ARN [34].
Anterior segment imaging (e.g., in vivo confocal microscopy)	Characterization of corneal and anterior segment changes	Confocal microscopy demonstrates subbasal nerve loss and inflammatory changes in HZO and supports the concept of corneal denervation; these techniques are mainly adjunctive and used in selected cases [31].
Aqueous or vitreous PCR	Etiologic confirmation, especially in posterior segment or atypical disease	Detection of VZV DNA in aqueous or vitreous samples is the diagnostic gold standard for ARN and PORN and distinguishes VZV from herpes simplex virus and cytomegalovirus [56,77].
Intraocular antibody testing (Goldmann–Witmer coefficient)	Supportive diagnosis in late or PCR-negative disease	Demonstrates intraocular production of VZV-specific IgG and is most useful in subacute or chronic presentations when viral DNA is no longer detectable but clinical suspicion remains high [77].
CNS evaluation (CSF PCR and intrathecal anti-VZV IgG)	Evaluation of optic neuritis, meningitis, encephalitis, or vasculopathy	CSF PCR and intrathecal VZV IgG synthesis support the diagnosis of CNS involvement in patients with neuro-ophthalmic or neurological symptoms [32].

Abbreviations: VZV, varicella-zoster virus; HZO, herpes zoster ophthalmicus; V1, ophthalmic division of the trigeminal nerve; OCT, optical coherence tomography; ARN, acute retinal necrosis; PORN, progressive outer retinal necrosis; CNS, central nervous system; CSF, cerebrospinal fluid; IgG, immunoglobulin G.

**Table 3:** Therapeutic approaches for varicella-zoster virus-related ocular disease.

Therapeutic Modality	Main Indication	Practical Considerations	Typical Duration
Oral antivirals (acyclovir, valacyclovir, famciclovir)	Acute HZO and most anterior segment manifestations in immunocompetent patients	Should be started as early as possible, ideally within 72 h of rash or ocular symptom onset; longer or prophylactic regimens may be considered in recurrent or high-risk disease according to clinical judgment and emerging evidence [15].	7–10 days for acute HZO [15]; weeks to months in recurrent or high-risk cases
Intravenous antivirals (acyclovir; foscarnet in selected cases)	Severe or sight-threatening disease (e.g., ARN, PORN), disseminated VZV, or infection in immunocompromised patients	Intravenous acyclovir is standard and is usually followed by a prolonged course of oral therapy; foscarnet is reserved for suspected acyclovir-resistant infection or profound immunosuppression and requires close renal and electrolyte monitoring [77].	Typically 10–14 days intravenously, followed by oral step-down therapy [118]
Intravitreal antivirals (e.g., foscarnet, ganciclovir)	Adjunctive treatment in necrotizing herpetic retinitis	Administered in combination with systemic antivirals to rapidly achieve high intraocular drug levels; may improve local disease control but carries procedural risks and is not a replacement for systemic therapy [56,119].	Repeated injections over days to weeks, depending on clinical response [120]
Topical corticosteroids	Immune-mediated stromal keratitis, disciform keratitis, endotheliitis, and anterior uveitis	Always combined with adequate antiviral therapy; effective in reducing inflammation and preventing synechiae but require gradual tapering and careful monitoring of IOP and cataract formation; not used for active epithelial keratitis with epithelial defects [15].	Several weeks to months, with slow tapering [121]
Systemic corticosteroids	Severe scleritis, optic neuritis, cranial neuropathies, orbital inflammation, and selected vasculitides	Introduced only after antiviral therapy has been initiated; high-dose oral or intravenous regimens may be used for a limited period with individualized tapering; carry a risk of exacerbating viral replication if used without antiviral cover [15,77].	Short-term (typically weeks), followed by gradual taper [122]
Adjunctive and surgical treatments	Management of complications and structural sequelae	Include cycloplegics for pain relief and synechiae prevention, glaucoma medications for secondary IOP elevation, and surgical options such as tarsorrhaphy or amniotic membrane transplantation for severe neurotrophic keratopathy and barrier laser or pars plana vitrectomy for high-risk acute retinal necrosis-related retinal detachment [15,80].	Highly case-dependent; often long-term or permanent management [15]

Abbreviations: VZV, varicella-zoster virus; HZO, herpes zoster ophthalmicus; ARN, acute retinal necrosis; PORN, progressive outer retinal necrosis; IOP, intraocular pressure.

## Data Availability

Not applicable.
